# Kynurenic acid is a potential overlapped biomarker between diagnosis and treatment response for depression from metabolome analysis

**DOI:** 10.1038/s41598-020-73918-z

**Published:** 2020-10-08

**Authors:** Hisayuki Erabi, Go Okada, Chiyo Shibasaki, Daiki Setoyama, Dongchon Kang, Masahiro Takamura, Atsuo Yoshino, Manabu Fuchikami, Akiko Kurata, Takahiro A. Kato, Shigeto Yamawaki, Yasumasa Okamoto

**Affiliations:** 1 Department of Psychiatry and Neurosciences, Graduate School of Biomedical Sciences, Hiroshima University, 1-2-3 Kasumi, Minami-ku, Hiroshima, 734-8551 Japan; 2 Department of Clinical Chemistry and Laboratory Medicine, Graduate School of Medical Sciences, Kyushu University, 3-1-1 Maidashi Higashi-Ku, Fukuoka, 812-8582 Japan; 3 Department of Neuropsychiatry, Graduate School of Medical Sciences, Kyushu University, 3-1-1 Maidashi Higashi-Ku, Fukuoka, 812-8582 Japan

**Keywords:** Metabolomics, Diagnostic markers, Predictive markers, Depression

## Abstract

Since optimal treatment at an early stage leads to remission of symptoms and recovery of function, putative biomarkers leading to early diagnosis and prediction of therapeutic responses are desired. The current study aimed to use a metabolomic approach to extract metabolites involved in both the diagnosis of major depressive disorder (MDD) and the prediction of therapeutic response for escitalopram. We compared plasma metabolites of MDD patients (n = 88) with those in healthy participants (n = 88) and found significant differences in the concentrations of 20 metabolites. We measured the Hamilton Rating Scale for Depression (HRSD) on 62 patients who completed approximately six-week treatment with escitalopram before and after treatment and found that kynurenic acid and kynurenine were significantly and negatively associated with HRSD reduction. Only one metabolite, kynurenic acid, was detected among 73 metabolites for overlapped biomarkers. Kynurenic acid was lower in MDD, and lower levels showed a better therapeutic response to escitalopram. Kynurenic acid is a metabolite in the kynurenine pathway that has been widely accepted as being a major mechanism in MDD. Overlapping biomarkers that facilitate diagnosis and prediction of the treatment response may help to improve disease classification and reduce the exposure of patients to less effective treatments in MDD.

## Introduction

The pathomechanism of major depressive disorder (MDD) remains largely unknown. Although there have been many studies to identify biomarkers for the diagnosis of MDD, there are currently no diagnostic biomarkers that are routinely used in clinical practice^1^. Furthermore, although there is a wide variety of treatment options for MDD, only approximately 40% of MDD patients achieve remission after initial treatment^2^.

Selective serotonin reuptake inhibitors (SSRIs) are commonly used as first-line treatment for MDD^3^. They are thought to increase the extracellular availability of the neurotransmitter serotonin by limiting its reabsorption into presynaptic cells, increasing serotonin levels in the synaptic cleft, and making it available for postsynaptic receptor binding^4^. However, only about half to two-thirds of patients respond to SSRIs, requiring weeks of treatment before an optimal therapeutic response is achieved^5^. The effects of antidepressants vary significantly from person to person, and finding the right drug at the right dose requires trial and error^6^. Clinical manifestations are insufficient to guide appropriate treatment options, and it is essential to develop biomarkers of MDD that will lead to the diagnosis of MDD or to predict response to treatment.

Biomarkers can be classified into four types as follows: diagnostic, predictive, prognostic, and therapeutic response. Diagnostic biomarkers can detect disease early and indicate future onset by non-invasive methods. Predictive biomarkers allow the identification of patients who are likely to benefit from therapy. Prognostic biomarkers provide information about disease course and outcome. Therapeutic response biomarkers predict the effect of therapeutic intervention and can be used as selection of efficacious antidepressant for MDD with some biological features^7,8^. Since optimal treatment at an early stage leads to remission of symptoms and recovery of function^9^, putative biomarkers leading to early diagnosis and prediction of therapeutic responses are desired.

In recent years, metabolomics approaches have attracted attention due to new possibilities for biomarkers. Metabolites are the final phenotype and are thought to be influenced by genetic and environmental factors and associated with disease pathology. The metabolomic mass spectrometry-based approach is a means of exhaustively searching for changes in vivo metabolites that are unpredictable from previous knowledge using unbiased techniques^8,10^. Recent advances in analytical chemistry have made this approach possible.

Various biological fluids, such as urine, plasma, and cerebrospinal fluid, have been analyzed. Blood in particular is easy and less invasive to obtain. One of the first metabolomics studies using blood in the field of MDD diagnosis was conducted by Paige et al. (2007), who analyzed approximately 800 metabolites in plasma in three groups as follows: people with depression, people in remission, and control participants. The depression group showed a significant overall decrease in gamma-aminobutyric acid (GABA) and medium-chain fatty acid levels^11^. Since then, many studies have been conducted on MDD metabolites^12–15^. According to MacDonald et al. (2019), nine diagnostic biomarkers have been identified in plasma of MDD patients, with glutamate and alanine showing up-regulation, and myo-inositol, GABA, phenylalanine, creatine, methionine, oleic acid, and tryptophan showing down-regulation^16^.

Kaddurah-Daouk et al. (2011) conducted one of the first metabolomic studies of treatment prediction for MDD. This study demonstrated the potential of metabolomics to provide information on the early efficacy of sertraline^17^ and another study (2013) reported that good therapeutic outcomes of MDD were associated with low levels of branched-chain amino acids^18^. Zhu et al. (2013) demonstrated that high pretreatment levels of 5-methoxytryptamine were associated with sertraline responsiveness^19^. Rotroff et al. (2016) demonstrated that none of the baseline metabolomes was significantly associated with treatment response to ketamine, esketamine, or placebo^20^. For citalopram and escitalopram treatment response, Bhattacharyya et al. (2019) suggested that higher baseline serotonin and 3-methoxy-4-hydroxyphenylglycol levels were associated with better responses to SSRIs^21^.

Although the knowledge about diagnostic biomarkers of MDD is expanding through this metabolomic approach, predictive biomarkers of antidepressant therapy are not enough and require further study. Besides, if a biomarker capable of simultaneously performing MDD diagnosis and treatment prediction can be established, treatment for MDD can be introduced more simply and efficiently. Based on the above points of view, the present study aims to extract markers that overlap diagnostic biomarkers of MDD and predictive biomarkers of treatment of escitalopram from many metabolites obtained by metabolomics. Escitalopram was selected because it has a highly selective, dose-dependent inhibitory effect on the serotonin transporter, is highly effective and well-tolerated, and the initial dosage is effective for treating depression^22^.

## Results

### Sample demographics

Demographic information including gender, age, as well as HRSD score, comorbidity of other mental disorders, and use of benzodiazepines for the study participants are shown in Table 1. MDD participants and healthy controls (HC) did not differ significantly in gender (42/88 (48%) female vs. 48/88 (55%) female, *p* = 0.37, chi-square test), or age (42.9 ± 12.2 vs. 41.3 ± 12.7, *p* = 0.42, two-sample t-test) at baseline. The average baseline HRSD rating was 19.3 ± 5.0. Forty-three patients (48.9%) had the first episode, and 34 patients (38.6%) had other psychiatric disorders. Thirteen patients (14.8%) were receiving benzodiazepines.

**Table 1:** Demographic and clinical characteristics of the patients with major depressive disorder (MDD) and healthy controls (HC).

	HC (n = 88)	MDD (n = 88)	*p*-value
Gender (male: female)	40/48	46/42	0.366
Age (years)	41.3 ± 12.7	42.9 ± 12.2	0.415
HRSD score at baseline		19.3 ± 5.0	
Episode (single: recurrent)		43/45	
Other mental disorder (yes: no)		34/54	
Use of benzodiazepines at baseline (yes: no)		13/75	

As for treatment response data, we excluded 26 patients due to withdrawal (n = 9), change to or combination with other antidepressants (n = 12), or discontinuation of escitalopram (n = 5). Thus, these data were obtained in 62 patients after approximately 6 weeks of treatment with escitalopram. Subjects were evaluated for at least 6 weeks and up to 8 weeks, and the mean duration of treatment was 45.9 ± 4.5 days. The initial dose was 5–10 mg and the dose after 6 weeks were 12.8 ± 5.1 mg (maximum total dose 13.7 mg/day). The clinical features are shown in Table 2. Follow-up HRSD was recorded for all 62 patients, and there was a significant difference in the HRSD scores recorded at baseline and follow-up (*p* < 0.001, paired-sample t-test). Of these, 34 patients (54.8%) received benzodiazepine concurrently. Twenty-nine patients (46.8%) showed a clinical response (≧ 50% reduction in the HRSD score), and 24 (38.7%) reported remission (follow-up HRSD ≦ 7).

**Table 2:** Demographic and clinical characteristics of the patients with major depressive disorder (MDD).

	Patients with MDD (n = 62)
Gender (male: female)	33/29
Age (years)	43.5 ± 12.9
HRSD score at baseline	19.1 ± 5.3
HRSD score after 6 weeks	10.5 ± 6.5
Dose of escitalopram at 6 weeks	12.8 ± 5.1
Maximum dose of escitalopram	13.7 ± 5.2
Use of benzodiazepine (yes: no)	34/28
Rate of patients with response (%)	46.8
Rate of patients with remission (%)	38.7

### Detection of diagnostic biomarkers

To detect metabolites useful for the diagnosis of MDD, we performed a metabolomic analysis of blood samples from 88 patients and 88 controls using LCMS-8060 in baseline plasma. Some of the present data have been overlapped with our previous report^39^. Seventy-three metabolites were identified, and blood concentrations were compared in both groups using the Mann–Whitney U test. Five metabolites, 5-oxoproline, 3-hydroxybutyrate, nicotinamide, glutamate and Putrescine, were significantly increased in MDD patients relative to HC. Moreover, the levels of 15 metabolites, sarcosine, serine, alanine, xanthurenate, xanthosine, tyrosine, phenylalanine, 3-methylhistidine, asparagine, kynurenic acid, 2-aminoisovaleric acid, threonine, tryptophan, pyruvate and 3-hydroxykynurenine, were significantly decreased in MDD patients relative to HC (Table 3).

**Table 3:** Plasma levels of baseline metabolites.

Direction	Label	HC mean	SD	MDD mean	SD	*p*-value Unc	FDR
HC < MDD	5-Oxoproline	1,152	882	2,435	2,114	0.000	0.001
	3-Hydroxybutyrate	8,644,814	505,743	8,865,845	421,071	0.000	0.007
	Nicotinamide	11,541	6,154	13,217	5,260	0.002	0.012
	Glutamate	6,919	9,018	11,994	13,212	0.009	0.036
	Putrescine	1,595	303	1,706	277	0.013	0.049
HC > MDD	Sarcosine	69,453	16,548	61,174	13,581	0.000	0.005
	Serine	92,105	24,072	79,291	15,941	0.000	0.006
	Alanine	82,149	19,181	72,960	16,844	0.000	0.007
	Xanthurenate	2,560	1,251	1,996	1,048	0.001	0.008
	Xanthosine	12,633	4488	10,799	3,600	0.001	0.008
	Tyrosine	111,690	23,698	100,114	22,465	0.001	0.008
	Phenylalanine	4,807	1,525	4,035	1,420	0.002	0.012
	3-Methylhistidine	43,625	53,005	23,761	31,546	0.002	0.013
	Asparagine	423	152	351	119	0.002	0.013
	Kynurenic acid	3,565	2,249	2,597	2,081	0.003	0.017
	2-Aminoisovaleric acid	48,355	11,111	43,648	10,695	0.004	0.020
	Threonine	1,223	666	953	493	0.006	0.027
	Tryptophan	19,537	6,102	17,262	6,302	0.009	0.036
	Pyruvate	21,199	8,482	17,996	7,431	0.008	0.036
	3-Hydroxykynurenine	1,166	424	1,011	411	0.008	0.036

### Investigation of predictive biomarkers

To detect metabolites useful for the treatment of escitalopram, we performed metabolome analysis on the metabolites in plasma of 62 patients who completed escitalopram treatment for about 6 weeks. Regression models were developed to identify metabolites involved in therapeutic response. Two metabolites, kynurenic acid and kynurenine, showed a significant negative correlation with the reduction rate of HRSD (Table 4). To control for the possibility that the results were affected by clinico-demographic characteristics, we repeated our analyses using multiple regression models that included gender, age, and baseline HRSD score as factors. The results were unaffected and both kynurenic acid and kynurenine showed a significant ability to predict the therapeutic response (kynurenic acid $\widehat{\beta }$= − 0.360, t =  − 2.911, *p* = 0.005; kynurenine $\widehat{\beta }$= − 0.435, t =  − 3.606, *p* = 0.001). The effects of age, gender, and baseline HRSD score were nonsignificant in both analyses. Since a significant relationship was found between metabolites and the reduction of HRSD scores (continuous variable), i.e., lower levels of kynurenic acid and kynurenine were associated with a better therapeutic response to escitalopram, we conducted additional analysis using a categorical variable (escitalopram responders vs. non-responders) to support this conclusion. The levels of these two metabolites were significantly lower in responders (≧ 50% reduction in HRSD score, n = 29) relative to non-responders (n = 33) at baseline (*p* = 0.002, *p* = 0.006, respectively).

**Table 4:** Correlation between reduction in Hamilton Rating Scale for Depression (HRSD) and plasma concentration.

Label	Spearman rho	*p*-value unc	FDR
Kynurenic acid	− 0.44	0.000	0.028
Kynurenine	− 0.43	0.001	0.020

### Overlap of diagnostic and therapeutic response biomarkers

**Figure 1:**
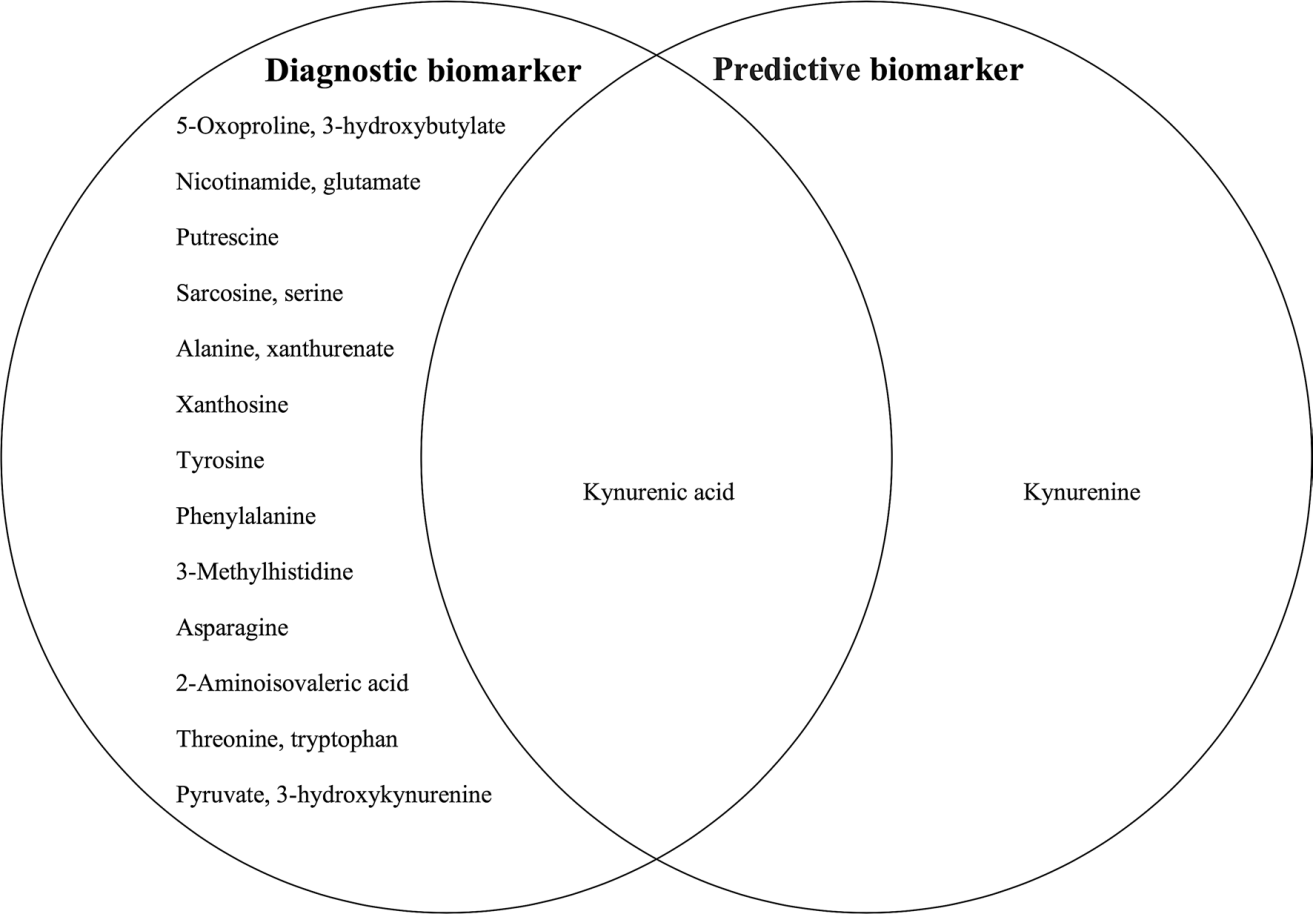
Venn diagram of diagnostic and predictive biomarkers. Twenty metabolites were identified as the diagnostic biomarker. Two metabolites were identified as predictive biomarker. Only kynurenic acid was identified as both diagnostic and predictive biomarker.

We examined the overlap of 20 different metabolites that distinguish MDD patients from HC and 2 metabolites that affect the prediction of escitalopram treatment response (overlapping biomarkers). As a result, among 73 metabolites initially detected, only kynurenic acid was overlapped as the candidate biomarkers for both diagnosis and treatment prediction (Fig. 1).

### Investigation of biomarkers in tryptophan-kynurenine pathway

Since only one metabolite, kynurenic acid, was detected among 73 metabolites as an overlapping biomarker, we investigated some metabolites in tryptophan-kynurenine pathway. In this pathway, we measured 4 metabolites, tryptophan (TRP), kynurenine (KYN), 3-Hydroxykynurenine (3-HK), kynurenic acid (KYNA). MDD patients had significantly lower levels of TRP, 3-HK, and KYNA than those in the HC group (Table 3). They also showed a lower level of KYN with a significant tendency (33,131 ± 23,967 for patients with MDD vs. 40,375 ± 23,460 for HC, uncorrected *p*-value = 0.023, false-discovery-rate (FDR) corrected *p* value = 0.074).

## Discussion

The current study used a metabolomic approach to identify plasma metabolites that could distinguish MDD patients from HC and predict the therapeutic response of escitalopram. It is the first study to investigate metabolites that overlap as diagnostic biomarkers of MDD and predictive biomarkers of escitalopram treatment response in the same group of patients. Of 73 metabolites initially detected, 20 broad-spectrum metabolites have been identified for diagnostic biomarkers, showing up-regulation of 5 metabolites, and down-regulation of 15 metabolites. Conversely, two metabolites have shown a significant negative correlation with treatment response for predictive biomarkers. Among these predictive biomarkers, two metabolites, KYN and KYNA, are members of the kynurenine pathway. For overlapping biomarkers, one metabolite, KYNA in the pathway was detected in the present study.

Metabolic factors in the kynurenine pathway have been widely accepted as being an essential mechanism in MDD. The kynurenine pathway begins with the conversion of TRP to KYN. Then, KYN continues to be metabolized mainly along two independent branches. For the first branch, KYN is transformed into 3-HK, 3-hydroxyanthranilic acid, and quinolinic acid (QUIN) by various enzymes. 3-HK and QUIN are N-methyl-d-aspartate (NMDA) receptor agonists, which have been demonstrated to exert neurotoxic effects^23,24^. For the second branch, KYN is transformed into KYNA by kynurenine aminotransferases. Increased serum KYNA is associated with aging, and reports of KYNA production independent of kynurenine aminotransferase are lacking; however, it is known to be partially formed from indole pyruvic acid non-enymatically^25^. KYNA is an NMDA receptor antagonist, which exerts a neuroprotective effect^26^.

Mounting evidence suggests that KYNA may be implicated in the pathophysiology of depression. The finding in the present study that mean plasma kynurenic acid levels were reduced in MDD patients relative to HC at baseline is consistent with previous literature. A recent review on metabolite levels of kynurenine pathway in patients with depression reported that out of the 17 studies on KYNA, 4 studies noted lower KYNA levels in patients with depression in comparison with HCs, with no differences found in KYNA levels between the groups in the remaining studies. A meta-analysis of these findings suggested that KYNA levels were decreased in patients with depression^27^. Another recent study also suggested that KYNA would be a potential biomarker in diagnosing depression patients^28^. In addition to reduced KYNA levels in MDD, a recent study demonstrated that the administration of KYNA induced antidepressant-like effects in an animal model of depression^29^.

Conversely, Halaris et al. (2015) assessed the metabolites of the tryptophan-kynurenine pathway among MDD patients before and after 8-weeks of escitalopram treatment. There was no change in the KYNA to KYNA/KYN ratio from baseline to week 8^30^. In their previous study, the significant increase of KYNA/KYN after the different medications was observed in the subgroup of patients with the first episode of depression^31^. Although, an association between lower baseline KYNA levels and better escitalopram response has not been previously reported, this finding appears compatible with the previous literature showing a beneficial effect of antidepressants on KYNA levels^32,33^. To our knowledge, no previous studies have used metabolomic analysis to examine overlapping metabolites as diagnostic biomarkers for MDD and predictive biomarkers for the treatment response to escitalopram in the same group of patients.

Several limitations must be noted in the current study. First, the sample size of the recruited participants was relatively small. Larger scale studies are needed for reproduction and validation. Second, all participants were recruited at the same site, resulting in the same ethnic group. Studies employing participants from different regions and institutions are needed to confirm whether they are replicated across regions and species. Third, for treatment response prediction, it is necessary to define groups of patients who may respond to the placebo to distinguish the effects that are specific to the active drug. Fourth, the results were limited to an early treatment response of up to 6 weeks. Longitudinal studies, including late evaluations after six weeks, are needed because drugs may be effective late. Fifth, renal function was not measured in this study and the effect of hypotensive drugs was not considered, although patients with serious physical disorders were excluded, and it was confirmed that no participants had a history of kidney disease or were undergoing treatment. Previous studies have suggested that kidney disease and concurrent drug treatments can affect KYN and KYNA levels^34,35^. In addition, this study did not consider the potential effects of diet and smoking, which may affect the concentrations of metabolites of the KYN pathway. Finally, the sensitivity and specificity of our results were relatively low, and metabolites alone may not be sufficient for biomarkers of depression. Combined use of neuroimaging with proteomic and metabolomic approaches could be more useful for facilitating the diagnosis and identification of subgroups related to the treatment response.

In conclusion, only one metabolite, KYNA, showed overlap as a potential biomarker for the diagnosis of MDD and prediction of response to treatment with escitalopram. KYNA was lower in MDD, with lower levels showing a better therapeutic response to escitalopram. An ideal biomarker can be detected at baseline and can be used to predict patients with an excellent or poor treatment response as well as a clinical diagnosis^36^. Identification of biomarker that are useful for both the diagnosis and treatment response may help to improve disease classification and reduce the exposure of patients to less effective methods in MDD. A similar approach of testing for specific biomarkers has resulted in changes in the classification and treatment of chronic myelogenous leukemia and lung cancer^37^.

## Methods

### Participants

All MDD patients were 25–75 years old and in the acute phase of MDD. They had not taken antidepressants for at least 1 month before entering the study, or their duration of antidepressant treatment was less than 5 days. They were screened using DSM-IV criteria for the diagnosis of MDD and MINI-International Psychiatric Structural Interview. The study excluded patients who had a diagnosis of current or previous psychotic disorder, current or past drug abuse, present high risk of suicide, and severe physical illness. Also excluded were pregnant or lactating women and patients who had used mood stabilizers, antipsychotics, central nervous system stimulants, or received electroconvulsive therapy within the past three months. A small number of patients were taking drugs to treat physical disorders, and five patients were taking antihypertensive drugs, but there was no concomitant renal dysfunction. On the day of blood sampling, the severity of depression at baseline was recorded using the 17-point HRSD.

The control group consisted of 88 healthy volunteers who were recruited from the community through newspaper advertisements and were confirmed to have no history of mental or physical disorders, pregnancy, or any medications or supplements. They were 20–75 years old and provided prior written informed consent. Structured clinical interviews were used to confirm that they had not experienced major depressive episodes in the past year. Individuals with a history of bipolar disorder or suicide attempt, or who had difficulty understanding the research objectives or filling out a self-report of a severe mental or physical disorder were excluded.

The experiments were carried out in accordance with the relevant guidelines and regulations. The Ethics Committee of Hiroshima University in Japan approved the current study. Prior written informed consent was obtained from all participants.

### Plasma sampling

Plasma metabolites were prepared as previously described^14,38,39^. A total of 100 ul (4 vol) of ice-cold methanol was added to 25 ul of plasma to extract water-soluble metabolites. The solution was vortexed, sonicated, and centrifuged (14,000 *g*, 4 °C, 15 min). Supernatants were collected and stored in 1.5 ml Eppendorf microtubes.

For amino acid extraction, 25 ul of plasma was added 0.1 100 M perchloric acid (4 vol), vortexed, sonicated, centrifuged (14,000 *g*, 4 °C, 15 min), and the supernatant was collected in 1.5 ml Eppendorf microtubes. For Liquid chromatograph-mass spectrometer (LC–MS) measurements, the obtained solution was diluted tenfold with each mobile phase, and a 5 ul solution (equivalent to 0.1 ul plasma) was applied.

### Metabolites analysis

Metabolites were analyzed as previously described^14,38,39^. LC–MS measurements were performed using LCMS 8060 instrument (Shimazu, Japan) as follows^14,38^. To measure various water-soluble metabolites, we separated the extracted solution on a Luna HILIC column 200A (150 mm × 2.0 mm, 3 mm, Phenomenex). The mobile phase consisted of 10 mM ammonium formate (A) and acetonitrile: 10 mM ammonium formate = 9: 1 (B). The gradient elution program was as follows: 0–2.5 min, 100%B; 2.5–4 min, 100–50%B; 4–7.5 min, 50–5%B; 7.5–10 min, 5%B; 10.1–12.5 min, 100%B. The flow rate was 0.3 mL/min, and the temperature of the column oven was 40 °C.

Another separation mode was used with an ACQUITY BEH Amide column (150 mm × 2.1 mm, 1.7 mm, water). The mobile phase consisted of 10 mM ammonium formate (A) and ten mM ammonium formate in acetonitrile (B). The gradient elution program was as follows: 0–2 min, 95%B; 2–5 min, 100–50%B; 5–8 min, 50%B; 8.1–11 min, 95%B. The flow rate was 0.4 mL/min, and the temperature of the column oven was 40 °C. The parameters for positive/negative electrospray ionization mode were as follows: drying gas flow rate, 15 L/min; nebulizer gas flow rate, 3 L/min; heating gas flow rate, 10 L/min; interface temperature, 300 °C; DL temperature, 250 °C; and heat block temperature, 400 °C; CID gas, 270 kPa.

To measure amino acids, we separated the extraction solution on an Intrada amino acid column (100 mm × 3.0 mm, 3 mm, Imtakt). The mobile phase consisted of 100 mM ammonium formate (A) and 0.1% formic acid in acetonitrile (B). The gradient elution program was as follows: 0–3 min, 75%B; 3–10 min, 75–0%B; 10–12.5 min, 0%B; 12.5–15 min, 75%B. The flow rate was 0.5 mL/min, and the temperature of the column oven was 40 °C. The parameters for positive/negative electrospray ionization mode were as follows: drying gas flow rate, 15 L/min; nebulizer gas flow rate, 3 L/min; heating gas flow rate, 10 L/min; interface temperature, 300 °C; DL temperature, 290 °C; and heat block temperature, 400 °C; CID gas, 270 kPa.

### Statistical analysis

We conducted a comparison analysis between MDD and HC with the Wilcoxon rank-sum test for each metabolite to determine those that could be used to distinguish patients from HC. We used a FDR correction by the Benjamini–Hochberg method to control for Type I error. In addition, we searched for a predictive biomarker of the treatment response after about 6-weeks of treatment by correlation analysis (Spearman’s rho) for each metabolite. The *p*-value of correlation analysis was adjusted by FDR as well. The result with a corrected *p*-value less than 0.05 was considered statistically significant. In addition, we performed multiple linear regression analysis for confirmatory purposes. In the regression model, treatment response was used as the dependent variable, and gender, age, baseline HRSD, and the level of each metabolite were used as independent variables. We estimated standardized partial regression coefficients ($\widehat{\beta }$) for each independent variable and tested for significance. The analyses were performed with MATLAB (version 2018b, MathWorks, Inc.). Lastly, we plotted receiver-operator response curves for both diagnostic biomarkers (MDD vs. HC classification) and treatment prediction biomarkers (responder vs. non-responder), and calculated the sensitivity, specificity, positive prediction value (PPV), and negative prediction value (NPV) at the cut-off score of the highest accuracy. The ROC analysis was performed with R software (version 4.0.2) and the ROCR package^40^.

## Supplementary information

Supplementary Information.
