# Supplementary material for: Kynurenic acid is a potential overlapped biomarker between diagnosis and treatment response for depression from metabolome analysis
Source: Sci Rep. 2020 Oct 8;10:16822. doi: 10.1038/s41598-020-73918-z (PMC7545168; doi:10.1038/s41598-020-73918-z)

Supplementary information for "Kynurenic acid is a potential overlapped biomarker between diagnosis and treatment response for depression from metabolome analysis".

Erabi H[1], Okada G[1], Shibasaki C[1], Setoyama D[2], Kang D[2], Takamura M[1], Yoshino A[1], Fuchikami M[1], Kurata A[1], Kato TA[3], Yamawaki S[1], Okamoto Y[1]

*oy@hiroshima-u.ac.jp

[1]Department of Psychiatry and Neurosciences, Graduate School of Biomedical Sciences, Hiroshima University, Hiroshima, Japan; [2]Department of Clinical Chemistry and Laboratory Medicine, Graduate School of Medical Sciences, Kyushu University, 3-1-1 Maidashi Higashi-Ku, Fukuoka 812-8582 Japan； [3]Department of Neuropsychiatry, Graduate School of Medical Sciences, Kyushu University, 3-1-1 Maidashi Higashi-Ku, Fukuoka 812-8582 Japan

Supplementary Information

Supplementary Methods

ROC analysis of the overlapping biomarker in predicting the diagnosis and treatment response.

Supplementary Results

Figure S1

Supplementary Methods

ROC analysis of the overlapping biomarker in predicting the diagnosis and treatment response.

ROC analysis was performed to evaluate the accuracy of the overlapping biomarker in predicting the diagnosis and treatment response. The predictive performance of kynurenic acid as a diagnostic and treatment prediction biomarker is shown in Figure S1.

Supplementary Results

Figure S1: ROC curves for Diagnosis and Treatment response results along with sensitivity, specificity, positive predictive value (PPV), negative predictive value (NPV), at the best cut-offs of the kynurenic acid.

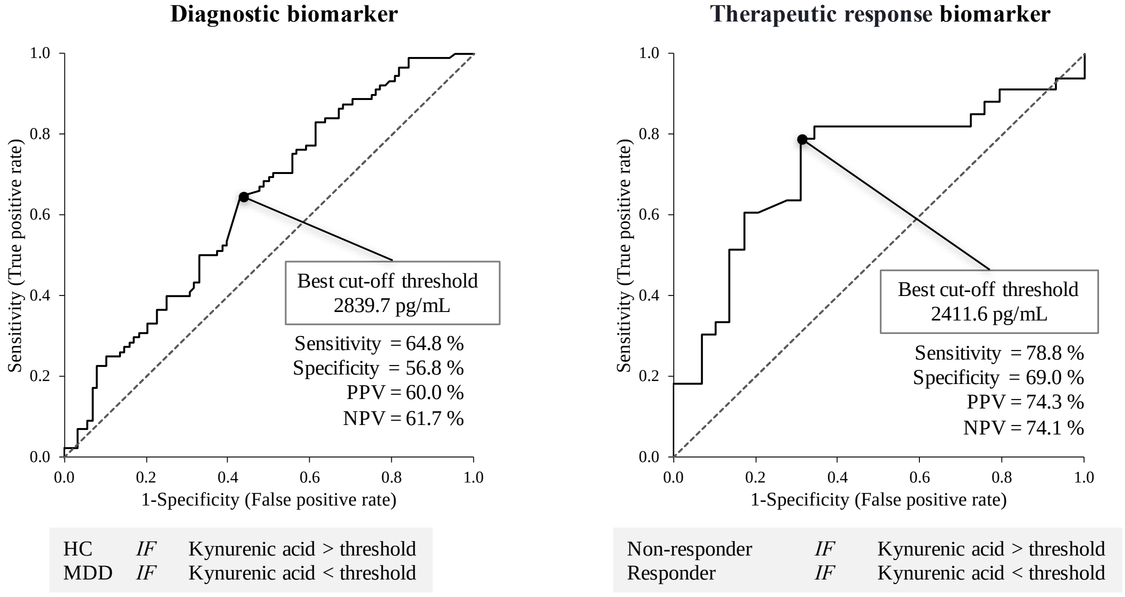

Supplement: Supplementary file 1 — Supplementary Information. [file 41598_2020_73918_MOESM1_ESM.pdf]
